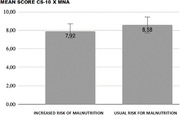# Do patients with early‐stage cognitive impairment have an increased risk to develop malnutrition? A cross‐sectional study

**DOI:** 10.1002/alz.090055

**Published:** 2025-01-09

**Authors:** Pedro de Castro Lopes, Amanda Aparecida Oliveira Leopoldino, João Carlos Barbosa Machado, Maira Tonidandel Barbosa, Luana Rodrigues Garcia, Bianca Pessoa Aguiar, Júlia Caroline Barbosa de Souza, João Pedro Neres Antunes Ferreira

**Affiliations:** ^1^ Rede Mater Dei de Saúde, Belo Horizonte, Minas Gerais Brazil; ^2^ Faculdade Ciências Médicas de Minas Gerais, Belo Horizonte, Minas Gerais Brazil

## Abstract

**Background:**

Malnutrition is a condition associated with negative outcomes in elderly patients, such as loss of functionality and mortality. The cause of malnutrition is multifactorial: secondary to changes in eating habits, dysphagia and loss of interest in food. It is a frequent condition in patients with advanced dementia. However, more studies are needed to deeply understand the relationship between nutritional status and cognition. The main objective of this study is to understand whether patients with early‐stage cognitive impairment have an increased risk for malnutrition, as well as patients with advanced dementia.

**Method:**

This is a cross‐sectional study in which 87 community‐dwelling elderly people were included and evaluated in relation to the presence of cognitive impairment and malnutrition. Patients with severe cognitive impairment, assessed by a score less than 6 in the CS‐10 (Point Cognitive Screening) were excluded. Individuals with a score between 6 and 7 on the CS‐10 were considered to have mild or moderate cognitive impairment and participants with a score greater than 7 on this test were considered to have no cognitive impairment. In order to evaluate risk of malnutrition, the Mini Nutritional Assessment (MNA) questionnaire was applied. Patients with a score lower than 12 were considered at risk of malnutrition and score higher than this was considered to be at no risk. In order to evaluate the association between the characteristics, Pearson’s chi‐square test was used. If the p‐value was lower than the significance level of 0.05, it is possible to conclude the association between a lower punctuation at MNA and a worse score in CS‐10.

**Result:**

Patients with alteration in MNA have an average score of 7.92 on the CS‐10. For patients who have not had any alteration in MNA, the mean score is 8.58 points. However, these differences are not significant (p‐value 0.088) and this is not a relevant association (p‐value 0.271).

**Conclusion:**

Despite of a tendency for patients with mild cognitive impairment to have a higher risk for developing malnutrition, it is not possible to affirm this relationship from this study, since no statistically significant association was identified.